# Hospital and Institutional News

**Published:** 1920-06-26

**Authors:** 


### HOSPITAL AND INSTITUTIONAL NEWS.

### THE KING'S FUND AND DR. ADDISON.

In reply to a question from Mr. Molone last week, Dr. Addison said that he was aware that two other hospitals beside the National Hospital for the Paralysed were closing in part. He added that the King's Fund was considering if interim or emergency assistance could be given Dr. Addison committed himself to nothing except a determination not to prejudice the voluntary principle. Since he added that he was considering how the hospitals could be helped, the inference is that he is waiting to see what the King's Fund finds to be practicable. For, *mutatis mutandis*, the help given by the King's Fund and projected by Dr. Addison is alike in principle. Neither can shoulder the whole burden, both can do something to relieve it. For the State to step in with assistance now would not be unlike the Fund deciding to give an emergency grant. The King's Fund, at all events, is the nearest approach to a common head or council for the hospitals of London.

### £250,000 FOR THE HOSPITALS OF LONDON.

Presumably the results of the inquiry made by Viscount Hambleden's Committee, appointed by the British Hospitals Association to ascertain the financial position of the London hospitals according to latest figures, have been supplied to King Edward's Hospital Fund, for use in allocating the £250,000 which, as was announced at Tuesday's meeting at St. James's Palace, is to be provided to carry the metropolitan hospitals through the crisis, or, rather, to stem the tide until some stable form of relief is devised. It is an excellent thing that these statistics, simple and rough and ready though they are, were gathered together in advance of the serious situation of the moment, when the quarterly bills are due for payment, rather than recourse should be made to the cumbrous, though valuable, returns required by the Central Funds in respect of the annual grants. The sum mentioned is one which will probably save the situation for the meantime, but, large though it is, will do no more. Moreover, as was pointed out at the King's Fund meeting, which is reported at length on page 328, the capital funds of King Edward's Fund are being encroached upon. As the annual distribution largely depends upon the interest on accumulated funds, this expedient was doubtless not decided upon without careful consideration, and, in the nature of things, is one that cannot be repeated indefinitely.

### THE HOSPITALS' PLATFORM.

The annual meeting of the British Hospitals Association, which is being held on Friday of this week at St. Thomas's Hospital, should be one of considerable interest. The activities of the Association during the past year have included the formation of a Scottish section, under Dr. D. J. Mackintosh, C.B., of Glasgow Western Infirmary, and of a London committee, which has been of valuable assistance to the King's Fund in its consideration of the hospitals' present financial position. A proposal will come before Friday's meeting to divide the country up into "Regions," with a view to the better representation of hospitals. Such a proposal is to be commended and, if carried into effect on well-organised lines, should increase interest in and add strength and vitality to the Association's work. In regard to the Scottish section of the Association, it is interesting to recall that the first conference of the British Hospitals Association, in whose reconstitution in 1910 Sir Henry Burdett was one of the prime movers, took place in Glasgow in September of that year. This conference was the forerunner of annual and other conferences in connection with the Association which have been held since in Manchester, Birmingham, Oxford, Newcastle-on-Tyne, and London.

### THE CAMBRIDGE MEETING.

At Cambridge the annual representative meeting of the British Medical Association begins in the Examination Hall on Friday, June 25, at 10 A.M. Sir Clifford Allbutt will give his presidential address to the Associates on the evening of Tuesday, June 29, in the Senate House. The scientific work of the meeting will be conducted in twelve sections, which meet in the New Museums on Wednesday, June 30; Thursday, July 1; and Friday, July 2. The morning will be devoted to discussions, and the afternoons to laboratory and clinical demonstrations. In our subsequent issues we hope to summarise some of the more important discussions bearing directly upon institutional work.

### A MEMORIAL TO SIR VICTOR HORSLEY.

A proposal is being made by the many friends of the late Sir Victor Horsley towards creating some form of permanent recognition of his services to medical science and a preliminary appeal has already appeared in the daily press. The endowment of a Victor Horsley lectureship has been suggested with the Senate of the University of London as its most appropriate trustee. The exact form of its scope and constitution will be considered by a committee of which Sir Charles Ballance is Chairman, Sir W. A. Arbuthnot Lane and Dr. E. J. Domville, Joint Honorary Secretaries, and Sir Frederick Mott and Dr. H. H. Tooth, Joint Treasurers. We read that the idea at present favoured is the foundation of an annual lecture, the subject of which is not necessarily confined to medicine and which may be delivered by any prominent man in any field of progress.

### THE RESEARCH DEFENCE SOCIETY.

As we go to press the annual general meeting of this Society is being held at 11 Chandos Street, the house of the Medical Society of London. The chair will be taken by Lord Lamington, the President, and Lord Knutsford, as Chairman of the Committee, will submit the annual report. The latter has pointed out that the Society has nowadays to reckon with a wider sort of attack from those hostile to research than has marked the earlier campaigns. The protective treatments, founded on and originating from the somewhat misunderstood animal experiments, are all likely in the near future to be impeded in their beneficial influence by a diversity of opposition which tends to insinuate itself between the new links forged between the sister sciences combining to form modern medicine. We read that the society, in appreciating the task before it and the variety of attacks it must now defeat, sets out "to educate the ignorant, to appease the fanatic, and to convince the reasonable man"—objectives that might well serve in connection with many a medical problem of to-day.

### THE AMERICAN HOSPITAL IN LONDON.

The scheme for founding an American hospital in London, decided upon at a meeting presided over by Lord Reading last year, is fast materialising, and we understand that practically all preparations in the United States are complete. No expense will be spared to make the institution one of the most efficient of its kind in the world, and the hospital will provide from its commencement treatment of patients of all nationalities. The post-graduate school it is intended to found will form a centre for American students and a rallying point for members of the medical profession in the United States when visiting these shores, where they will find special facilities for scientific study and research, in addition to those provided at English hospitals, which have always welcomed them as visitors. A dinner, in connection with the hospital scheme, is to be given next month to Dr. Charles Mayo, of the well-known Rochester clinic. Mr. Balfour will probably be one of the speakers, and a notable attendance of medical men is expected.

### QUACK MEDICINES.

A useful reference to the unsatisfactory control of quack medicines in this country is made in a recent number of the *Medical Press*. After describing the relatively efficient legal manner in which the American public is protected against the nostrums of quacks, the author goes on to describe how parlous, in comparison, is our own position upon this question. Again and again some concoction of the cheapest drugs is made up and flamboyantly advertised as a cure-all. The newspaper proprietors, who stand to gain greatly from the vast number of such advertisements, naturally oppose any legislative attempt to interfere with this business. Unfortunately, the wealthy newspaper-owners form a powerful body, and have the protection of political influence. It is true that some papers are exempt from this criticism, as in the case of *The Times*, which on June 7 headed its Personal column, "Advertisements of medical establishments or remedies are not accepted without satisfactory references." But the question involved is one of principle, and, unfortunately, that accepted in some quarters is one which, with our contemporary, we would most heartily condemn. It is a reasonable demand that Dr. Addison should give further attention to the Report of the Select Committee on Patent Medicines laid before the Houses of Parliament in 1914, and to his promise, through Lord Sandhurst, in the House of Lords in 1918, to bring in a Bill to give effect to that Report.

### WILL THE RELIEF FUND RELIEVE?

There is a feeling that after all there may be a chance of getting hold of part of the hoarded millions of the National Relief Fund to help the hospitals out of their pressing difficulties. The Government has always disclaimed any control or influence over the Fund which, managed by trustees, has always maintained that gifts to hospitals do not come within the objects. But the chief object is to relieve distress arising out of the war, and we cannot conceive any greater distress than would be caused should the voluntary hospitals shut their doors. It is stated that Dr. Addison has suggested grants from the Fund and that negotiations are proceeding with this object in view. If it is merely a question of helping the trustees to remove themselves from an untenable position, the ingenuity of our politicians should be able to contrive a smooth and graceful path.

### CONCERTED ACTION FOR THE THREE FUNDS.

Although we have already alluded to the plan for the concerted action of the Three Great Funds, it may be well to give the terms of the invitation issued by the Hospital Saturday Fund to its sister bodies. The letter sent to them is given in the current number of The Hospital Saturday Fund Journal. It says "the time has arrived for the institution of a united appeal and, for this purpose [the Saturday Fund], desires the co-operation of the British Red Cross Society, the King Edward's Hospital Fund, the League of Mercy, and the Hospital Sunday Fund. . . It is thought that a 'Hospital Week' might be inaugurated when *special* gifts and collections could be invited, so as not to interfere with the ordinary income of the respective organisations. All moneys received during this week could be paid into a central fund, and afterwards disbursed to the hospitals in accordance with the individual requirements." The appeal proposed is "a special one for a special emergency." The replies to the invitation, the *Journal* states, have been "very satisfactory, and it is hoped that a scheme on the lines suggested will shortly be adopted."

### THE REWARD OF METHOD AT WEST BROMWICH.

A hint to other hospitals comes from West Bromwich, where the hospital has begun to raise useful sums from sections of the public never previously approached. At a special meeting only the other day, Mr. A. Loach, chairman of the Hospital Saturday Committee, said that he had attended a meeting of the Tradesmen's Association, which had never subscribed before. Apparently the Association had never even been asked for a subscription. It resolved, however, to ask every tradesman in the town to contribute at least one guinea a year. Mr. J. E. Cox, the Mayor, said that the attention of tradesmen had never been called to the hospital. He expected that their contributions would amount to £200 or even £300. He further suggested that shop workers should be appealed to as the men in factories were. The tradespeople were willing, and some had adopted the system in vogue at different works. We note that the licensing trade, which subscribed last year £18, has now adopted a system of organised collection which is expected to yield £500.

### THE RED CROSS LIBRARY.

THE activities of the British Red Cross War Library in peace time will, it is hoped, distribute books and magazines beyond the limits of military and pensioners' hospitals; this is instanced by the expressed intention to provide story and picture books for the children in ordinary hospitals. In answer to an appeal by eminent workers in the military and naval hospitals in the Great War, the War Library has been reorganised, with headquarters at 48 Queen's Gardens, Lancaster Gate, W. 2, and the Committee have undertaken that no hospital in the United Kingdom which wishes for literature shall be without it. Books are provided on the basis of a book a bed, and special books are sent when patients ask for them—a source of considerable pleasure. Many who are unable in these hard times to give as much in cash as they would wish will easily be able to find amongst their surplus possessions an interesting book or magazine which will help to pass away an hour of sickness or convalescence.

### THE KENTISH FARMERS' DECISION.

A MEETING of representatives of the National Farmers' Union within the district served by the West Kent General Hospital has pledged itself to help to raise the £50,000 required from the country districts to complete the extension scheme. A hundred parishes outside Maidstone will take part in the campaign to be opened about the first week in October. Mr. Shirley Davis is the organising secretary, and Mr. G. Foster Clark and Mr. B. Champion moved and seconded the resolution. It is interesting that the population of the country districts is nearly twice that of Maidstone itself; and we are glad to add this effort of the Kentish farmers to those THE HOSPITAL has already described in Herefordshire and Suffolk. Farmers' funds for hospitals have not always been very actively supported for reasons some of which the new Bishop of Durham explained before his translation from Hereford. The hospitals now need organised support from every section. This organisation hitherto has been strongest in the towns, but it is encouraging to see the rural districts coming into line with them.

### THE DUKE OF PORTLAND'S APPEAL.

THE financial troubles of the ordinary voluntary hospitals are not unshared by the hospitals for incurables, which are popularly, and erroneously, supposed to get so much in the shape of legacies that it takes but little effort in the way of begging to obtain the rest of the required revenue. The British Home and Hospital for Incurables at Streatham is greatly in need of friends at the present time, and particularly addresses itself to those who can feel for middle-class people, whose sturdy independence renders them inarticulate when in need of help or asylum during chronic illness. The Streatham Home is committed to an annual expenditure of £25,000, and its only reliable yearly income is £5,000. The sum of £10,000 is owing to the bankers, and £50,000, the Duke of Portland states in his appeal, is required to re-establish the work upon a sound financial basis. It is stated that the institution is in real danger of having to close its doors because of inadequate support.

### PATIENTS ENTERTAIN THEIR COMMITTEE.

WITHIN the limits of our memory the recent entertaining of the trustees of the Treloar Cripples' Home by the Old Boys' Association is unique. The occasion was a dinner at Anderton's Hotel, when Sir Henry J. Gauvain, to whose skill as a surgeon in many cases must be due the fact that the Old Boys are Old Boys, presided at the function, and was supported by Sir William Treloar, the Bishop of London, Mr. Pett Ridge (who knows not a little about boys), Sir William Dunn, and others. Perhaps the most touching feature of the gathering was the presentation of a silver bowl to the Chairman, in recognition of the wonders he had worked for every Old Boy present. Mr. F. Hayden, one of the hosts, made a neat speech, on behalf of himself and others who had been set on their legs in more senses than one by the Cripples' Hospital and College at Alton, and said that the gathering represented ten years' work in the interests of those whose physical handicap would otherwise have kept them from being what they were to-day—able to earn their living and to be independent.

### DOCTORS AND MIDWIVES.

MIDDLESEX COUNTY COUNCIL reports a year's experience of the payment of fees to medical men called in by midwives. During 1919 the number of applications from medical practitioners for the payment of fees was 247. There were 927 notifications of sending for medical aid received from midwives. Hence it would appear that in 73 per cent. of cases in which a doctor had been summoned payment had been made to the doctor direct by the family, and that in only 27 per cent. of cases had there been any need for the doctor to apply to the County Council for his fee.

### THIS WEEK'S DRUG MARKET.

BUSINESS in the drug market continues extremely dull, and there is nothing to indicate that an improvement is pending. With prices tending downwards buyers are not disposed to commit themselves to large purchases, and the longer they can continue this policy the larger will stocks become, and the more likelihood will there be that prices will find a lower but steadier level. The downward movement is becoming more comprehensive, but here and there are articles which tend upwards in price while the values of a number of drugs are firmly maintained. In synthetic products generally there is an easier undercurrent, but nothing in the nature of a slump. Camphor, menthol, and Japanese pepermint oil have again moved downwards.

